# Pre-defined gene co-expression modules in rheumatoid arthritis transition towards molecular health following anti-TNF therapy

**DOI:** 10.1093/rheumatology/keac204

**Published:** 2022-04-04

**Authors:** Megan Sutcliffe, Nisha Nair, James Oliver, Ann W Morgan, John D Isaacs, Anthony G Wilson, Suzanne M M Verstappen, Sebastien Viatte, Kimme L Hyrich, Andrew P Morris, Anne Barton, Darren Plant

**Affiliations:** Versus Arthritis Centre for Genetics and Genomics, Division of Musculoskeletal Sciences, The University of Manchester; Versus Arthritis Centre for Genetics and Genomics, Division of Musculoskeletal Sciences, The University of Manchester; NIHR Manchester Biomedical Research Centre, Manchester University NHS Foundation Trust, Manchester Academic Health Sciences Centre, Manchester; Versus Arthritis Centre for Genetics and Genomics, Division of Musculoskeletal Sciences, The University of Manchester; NIHR Manchester Biomedical Research Centre, Manchester University NHS Foundation Trust, Manchester Academic Health Sciences Centre, Manchester; School of Medicine, University of Leeds & NIHR Leeds Biomedical Research Centre and NIHR In Vitro Diagnostic Co-operative, Leeds Teaching Hospitals NHS Trust, University of Leeds, Leeds; Translational & Clinical Research Institution, Newcastle University & Musculoskeletal Unit, Newcastle upon Tyne Hospitals NHS Foundation Trust, Newcastle University, Newcastle upon Tyne, UK; School of Medicine & Medical Science, Conway Institute, University College Dublin, Bellfield, Dublin 4, Ireland; NIHR Manchester Biomedical Research Centre, Manchester University NHS Foundation Trust, Manchester Academic Health Sciences Centre, Manchester; Versus Arthritis Centre for Epidemiology, Centre for Musculoskeletal Research; Versus Arthritis Centre for Genetics and Genomics, Division of Musculoskeletal Sciences, The University of Manchester; NIHR Manchester Biomedical Research Centre, Manchester University NHS Foundation Trust, Manchester Academic Health Sciences Centre, Manchester; Lydia Becker Institute of Immunology and Inflammation, Faculty of Biology, Medicine and Health, The University of Manchester, Manchester, UK; NIHR Manchester Biomedical Research Centre, Manchester University NHS Foundation Trust, Manchester Academic Health Sciences Centre, Manchester; Versus Arthritis Centre for Epidemiology, Centre for Musculoskeletal Research; Versus Arthritis Centre for Genetics and Genomics, Division of Musculoskeletal Sciences, The University of Manchester; Versus Arthritis Centre for Genetics and Genomics, Division of Musculoskeletal Sciences, The University of Manchester; NIHR Manchester Biomedical Research Centre, Manchester University NHS Foundation Trust, Manchester Academic Health Sciences Centre, Manchester; Versus Arthritis Centre for Genetics and Genomics, Division of Musculoskeletal Sciences, The University of Manchester; NIHR Manchester Biomedical Research Centre, Manchester University NHS Foundation Trust, Manchester Academic Health Sciences Centre, Manchester

**Keywords:** Biomarkers, RA, biological therapies, inflammation, genetics, treatment response, TNFi, transcriptomics

## Abstract

**Background:**

No reliable biomarkers to predict response to TNF inhibitors (TNFi) in RA patients currently exist. The aims of this study were to replicate changes in gene co-expression modules that were previously reported in response to TNFi therapy in RA; to test if changes in module expression are specific to TNFi therapy; and to determine whether module expression transitions towards a disease-free state in responding patients.

**Method:**

Published transcriptomic data from the whole blood of disease-free controls (*n* = 10) and RA patients, treated with the TNFi adalimumab (*n* = 70) or methotrexate (*n* = 85), were studied. Treatment response was assessed using the EULAR response criteria following 3 or 6 months of treatment. Change in transcript expression between pre- and post-treatment was recorded for previously defined modules. Linear mixed models tested whether modular expression after treatment transitioned towards a disease-free state.

**Results:**

For 25 of the 27 modules, change in expression between pre- and post-treatment in the adalimumab cohort replicated published findings. Of these 25 modules, six transitioned towards a disease-free state by 3 months (*P* *<* 0.05), irrespective of clinical response. One module (M3.2), related to inflammation and TNF biology, significantly correlated with response to adalimumab. Similar patterns of modular expression, with reduced magnitude, were observed in the methotrexate cohort.

**Conclusion:**

This study provides independent validation of changes in module expression in response to therapy in RA. However, these effects are not specific to TNFi. Further studies are required to determine whether specific modules could assist molecular classification of therapeutic response.

Rheumatology key messages

- In the TNFi cohort, expression of 25 of the 27 modules mirrored published findings.
- Reproducible changes in module expression were observed regardless of treatment response in RA patients.
- One module (M3.2, inflammation related) appeared to be dependent on good response to adalimumab.

## Introduction

Despite the wealth of treatment options currently available to RA patients, none are universally effective. RA patients are initially treated with conventional synthetic DMARDs (csDMARDs) such as MTX. Where first-line therapy is inadequate in controlling inflammation, patients are prescribed a biological DMARD (bDMARD), often in combination with MTX [1–3]. The most commonly prescribed group of bDMARDs are TNF inhibitors (TNFi) [3–6].

In the UK, treatment response to bDMARDs is determined 3 to 6 months following treatment initiation, according to the National Institute for Health and Care Excellence (NICE) guidance [7]. Although successful in treating RA, bDMARDs are ineffective for ∼40% of patients, and treatment switching is recommended in non-responding patients by 3 or 6 months. However, data from the British Society for Rheumatology Biologics Registry (BSRBR-RA) showed that a significant proportion of patients are cycling through a high number of bDMARDs, with over 20% trying three or more bDMARDs, and 30% trying two or more distinct classes of bDMARDs to control disease without significant adverse events [8].The current trial-and-error pathway means that a large minority of patients are treated sub-optimally for many months or even years [1, 9, 10].

Because radiographic damage caused by uncontrolled inflammation can occur rapidly [11], swift decisions about therapy changes should improve patient outcomes. The discovery of reliable biomarkers of TNFi response would aid more informed treatment strategies, including objective monitoring and the development of predictive tests, thus improving patient prognosis [11, 12]. However, to date, biomarker studies of treatment response in RA have not replicated across studies and populations [4].

One notable exception is a transcriptomic study that reported reproducible findings when investigating changes in 27 pre-defined gene co-expression modules in good responders and non-responders to TNFi therapy, across three independent RA cohorts recruited in the USA [13].

Gene co-expression modules were previously defined by Chaussabel *et al.* [14], following the analysis of 239 peripheral blood mononuclear cell (PBMC) samples obtained from individuals with systemic autoimmune diseases, cancers, microbial infections and liver transplant recipients undergoing immunosuppressive therapy. Modules were characterized after assessing transcript clustering patterns, in addition to identifying functional associations amongst transcripts and genes that were frequently co-expressed in disease.

Using the same gene co-expression modules, Oswald *et al.* identified module expression in RA patients consistently changed in good responders after 3 months of treatment with a TNFi, but fewer changes were observed in TNFi non-responders [13]. In the current study, we aimed to determine: (i) whether modular changes in gene expression during early treatment with a TNFi are observed in UK-recruited RA patient samples; (ii) whether the effects are drug-specific, by investigating a MTX-treated cohort; and (iii) whether gene expression modules transition towards a disease-free state in responding patients.

## Methods

### Patient cohort

Two prospective RA patient study cohorts and 10 disease-free controls were studied. One RA cohort was treated with adalimumab, and the other RA cohort was treated with MTX. Summary demographic information on both patient cohorts and disease-free controls are shown in Table 1.

**Table 1:** Baseline characteristics in the two RA cohorts studied, and the disease-free controls

Adalimumab cohort			
Characteristic	Good-responders (*n* = 50)	Non-responders (*n* = 20)	*P*-value
Age, mean (s.d.)	58.1 (13.1)	55.3 (13)	0.42^a^
Female, *n* (%)	31 (62)	15 (75)	0.30^b^
Baseline DAS28, mean (s.d.)	5.07 (0.90)	5.09 (0.90)	0.93^a^
Baseline HAQ score, median (IQR)	1.5 (1, 2.13)	1.8 (1.4, 2.3)	0.21^c^
Concurrent DMARD therapy, *n* (%)	46 (92)	15 (75)	0.06^b^

Methotrexate cohort			
Characteristic	Good-responders (*n* = 42)	Non-responders (*n* = 43)	*P*-value
Age, mean (s.d.)	59 (15)	55 (14)	0.28^a^
Female, n (%)	32 (76)	33(77)	0.95^b^
Baseline DAS28, mean (s.d.)	4.8 (1.0)	4.0 (1.3)	0.001^a^
Baseline HAQ score, median (IQR)	1.18 (0.9, 1.7)	1.0 (0.3, 1.6)	0.07^c^
Disease-free controls			
Age, mean (s.d.)	48 (7.4)		
Female, *n* (%)	7 (70)		

DAS28: 28-joint count DAS; concurrent DMARD therapy: concurrent treatment with MTX; IQR: interquartile range.

*P*-value;

a two-sample *t* test,

b chi-squared test,

c Wilcoxon rank-sum test.

The adalimumab cohort (*n* = 70) were recruited from UK centres to the Biologics in Rheumatoid Arthritis Genetics and Genomics Study Syndicate (BRAGGSS), previously described by Oliver *et al.* [15] Eligible patients were white adults with a clinician diagnosis of RA, and about to begin treatment with adalimumab for the first time for RA. The majority of patients (87%) were treated with a concurrent DMARD (including MTX). Patients were categorized as either good (*n* = 50) or non-responders (*n* = 20) to treatment following 3 months of therapy using established EULAR response criteria [16]. Non-responders were excluded if anti-drug antibodies were detected in serum samples by radioimmunoassay at 3 months and/or if they self-reported non-adherence. Ethics was approved by the North West 6 Central Manchester South Research Ethics Committee (COREC 04/Q1403/37) and all patients provided written consent [15].

The MTX cohort (*n* = 85) were recruited from the Rheumatoid Arthritis Medication Study (RAMS), a UK multicentre (*n* = 38 centres) one-year longitudinal observational study that enrolled new-onset RA patients who are about to commence therapy with MTX as their first csDMARD. Treatment response was assessed 6 months after treatment using the EULAR response criteria. Patients were then categorized as good (*n* = 42) or non-responders (*n* = 43) to MTX, as described above. RAMS was approved by the Central Manchester NHS Research Ethics Committee (reference 08/H1008/25) and all patients provided written informed consent [17, 18].

The disease-free controls were individuals without RA, recruited under the National Repository for Healthy Volunteers (NRHV) study within the Versus Arthritis Centre for Genetics and Genomics at the University of Manchester. Ethical approval was obtained (reference REC 99/8/84) and all volunteers gave written informed consent in compliance with Good Clinical Practice and the Declaration of Helsinki.

### Transcriptome measurement

In the adalimumab cohort, whole blood gene expression profiles were captured using the Affymetrix Human Transcriptome Array 2.0 (HTA) at pre-treatment and following 3 months of treatment. In the MTX cohort, Illumina HumanHT-12 v3 Array measured whole blood expression at pre-treatment and following 4 weeks of treatment.

### Statistical analysis

As two different arrays were used to acquire the transcript level gene expression data, specific packages that corresponded to each array were used to extract the raw transcript expression data. Pre-analysis quality control steps for the adalimumab and MTX-treated cohorts have been previously described [15, 18].

Briefly, all array files were processed using R (version 3.6.1). For HTA array data, the annotation package *pd.hta.2.0* was used for platform design. The *affy* package was used to summarize probe level data into a single expression value for each transcript, before transcripts were quantile normalized and log_2_ transformed. The *hta20transcriptcluster.db* and *biomaRT* packages were used to map Affymetrix probe identifiers to the corresponding Entrez gene identifier (Entrez ID). For HumanHT-12 array data, GenomeStudio software evaluated bead-level expression and the Bioconductor package, *limma* was used for quality control. Probes that were not expressed or mapped to more than one genomic location were removed. Data were then log_2_ transformed and quantile normalized. The *illuminaHumanv4.db* package was used to annotate transcripts with Entrez IDs.

The *PCAmethods* package was used to calculate principal components and to assess potential run order effects or outlier samples. The *limma* package was used to test for differential expression between pre-treatment and on-treatment time points in good and non-responders separately (e.g. change in transcription between baseline and 3 months in good responders to adalimumab). Statistical models included baseline DAS28, age, gender, concurrent DMARD use, HAQ scores, smoking habits (never, past or current smoker) and array weights (calculated using the *arrayWeights()* function) as fixed effects, and patient ID as a random effect as previously described [15, 18].

For both patient cohorts the log fold change, average expression, t-statistic and *P*-value returned from the *limma eBayes* function was stored for modular gene expression analysis.

### Modular analysis of transcriptome

Data from each cohort were analysed according to the methods described by Oswald *et al.* [13] using the 27 gene co-expression modules previously defined by Chaussabel *et al.* [14] and were numbered M1.1-M3.9. Modules were labelled in accordance with the identity of gene transcripts present in modules e.g. platelets, B cells, cytotoxic cells. Where modules were not easily characterized, no biological name was given and they remained defined as a number [13]. First, probe IDs were mapped to their corresponding Entrez ID and the transcriptomic data generated by Chaussabel *et al.* were also checked to ensure up-to-date Entrez IDs were mapped to the Affymetrix identifier in the original study [14].

For good- and non-responders, probes with a significant change in expression (*P*-value <0.05) were identified between pre- and post-treatment samples using *limma*. The proportion of significantly changed probes that had a positive or negative fold change in expression within each module was calculated. Contingency tables were computed for each module and a Fishers exact test determined which modules showed statistically significant changes in expression in good and non-responders, separately. The level of significance was corrected for multiple comparisons between the 27 modules using the Bonferroni correction algorithm (*P*-value <8.5e-03).

### Comparison of patients and disease-free controls

To test if changes in module gene expression in good- and non-responders had transitioned towards a disease-free state, comparisons were made between patients (at baseline and after treatment) and disease-free controls. The Wilcoxon Signed-Rank Test was used to determine whether the disease-free controls were sex-matched. Gene expression data from disease-free controls were pre-processed using the methods described for the adalimumab cohort. Batch effects between patients and disease-free controls were assessed using principal components analysis and then corrected for using the *ComBat* function within the *sva* package in R. No correlation between the first principal component and age, gender, DMARD use, baseline DAS28, DAS28 components, RIN, or RNA extraction batch Probes that significantly changed in expression between baseline and follow-up were identified. The *lme4* package was used to compare probe expression within each module using linear mixed models at baseline and after treatment, including a fixed-effect for patient/control status, and independent random effects for probe ID and patient ID. Finally, densities were plotted using *ggplot2* to visualize differences in baseline, follow-up and disease-free control module expression in the good and non-responders to adalimumab separately.

### Data availability

De-identified data presented in this manuscript are available via Figshare data using the following link: https://doi.org/10.48420/17061680.v1.

## Results

### Adalimumab cohort

Consistent with the findings from three independent US-recruited RA cohorts, previously described by Oswald *et al.* the expression of 25 of the 27 modules changed in the same direction in the UK-based adalimumab cohort. The expression of module M1.1 (plasma cells), M1.3 (B cells), M1.7 & M2.4 [major histocompatibility complex (MHC) and ribosomal proteins], M2.8 (T cells) and modules with no current nomenclature (M2.11, M2.7, M3.4, M3.6, M3.7, M3.8 and M3.9) significantly (*P* <8.5e-03) increased in expression between baseline and 3 months, in good- and non-responders. One module (M1.6) significantly increased (*P* <8.5e-03) in expression in the good responders, but not in non-responders.

Module M2.2 (neutrophil biology) significantly decreased in expression in good responders to adalimumab, but not in non-responders. Conversely, expression of module M2.9 significantly decreased in the non-responder group, while expression did not significantly change in good responders.

**Fig. 1:**
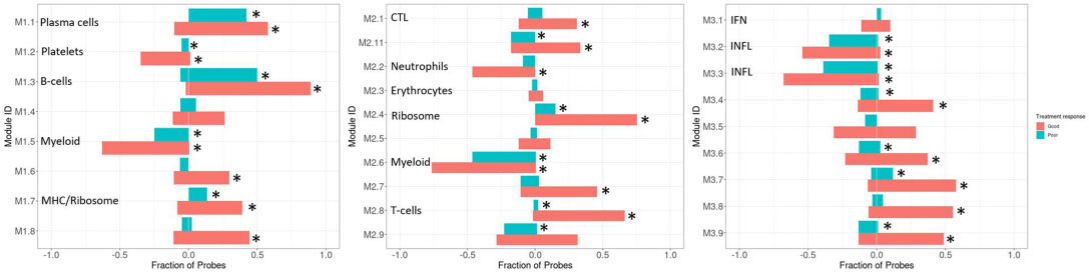
Change in module expression between baseline and follow-up in good and non-responders to adalimumab *A Fisher’s exact test was performed to test for statistical significance in the fraction of probes that significantly changed in expression between baseline and follow-up (3 months) in the good and non-responders to adalimumab (Bonferroni adjusted *P* <8.5e-03). From left to right, graphs show module 1.1–3.9. Red bars indicate good responders and green bars show non-responders to adalimumab. Direction of bars 0 to 1 indicates increase between pre-treatment and post-treatment and 0 to –1 indicates a decrease between pre-treatment and post-treatment. CTL: cytotoxic T lymphocyte; IFN: IFN inducible; INFL: inflammation.

Moreover, a significant fraction of probes in module M1.2 (platelets), M1.5 and M2.6 (myeloid cells) and M3.2 and M3.3 (inflammation) significantly decreased (*P* <8.5e-03) in expression between baseline and 3 months. These changes were observed regardless of adalimumab treatment response status by 3 months (Fig. 1 and Supplementary Table S1, available at *Rheumatology* online).

One module, module M2.7, did not reflect published findings, as between pre-treatment and 3 months, module expression significantly increased (*P* <8.5e-03) in good responders to adalimumab. However, no change in module M2.7 expression (*P* >0.005) was demonstrated in the three independent US-recruited cohorts reported by Oswald *et al.* [13]. The function of module M2.7 is currently undetermined.

Furthermore, four modules – module M2.3 (erythrocyte biology), M2.5, M3.1 (interferon inducibility) and M3.5 – did not significantly change in expression between baseline and 3 months in either good- or non-responder groups to adalimumab. This result mirrored published results by Oswald *et al.* as module M2.5 and M3.1 did not change in expression, whereas one of the US cohorts demonstrated significant changes in modules M2.3 and M3.5 (*P* *<*0.005) [13].

### MTX cohort

**Fig. 2:**
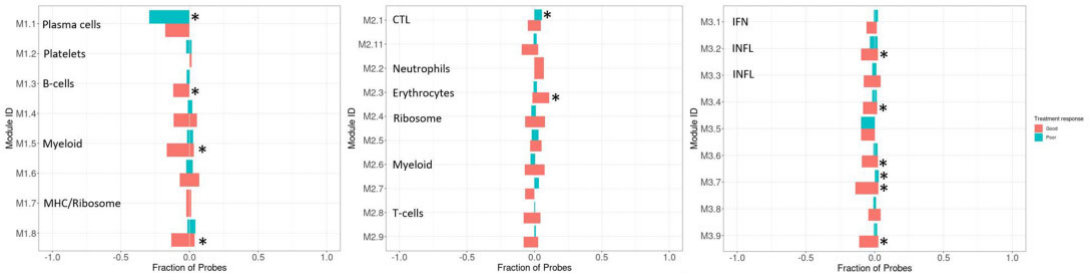
Change in module expression between baseline and follow-up in good and non-responders to methotrexate *A Fisher’s exact test was performed to test for statistical significance in the fraction of probes that significantly changed in expression between baseline and follow-up (4 weeks) in the good and non-responders to methotrexate (Bonferroni adjusted *P* <8.5e-03). From left to right, graphs show module 1.1–3.9. Red bars indicate good responders and green bars show non-responders to methotrexate. Direction of bars 0 to 1 indicates increase between pre-treatment and post-treatment and 0 to –1 indicates a decrease between pre-treatment and post-treatment. CTL: cytotoxic T lymphocyte; IFN: IFN inducible; INFL: inflammation.

Change in module expression was also assessed in RA patients whom had commenced treatment with MTX for the first time, where significant changes in modular expression were observed between baseline and 4 weeks (*P*-value <8.5e-03, Fig. 2 and Supplementary Table S2, available at *Rheumatology* online). One module (M2.3) significantly increased in expression (*P* <8.5e-03) with treatment in the good responders. Furthermore, module M2.1 (linked to cytotoxic T-cell biology) significantly increased in expression (*P* *<*8.5e-03) in the MTX non-responder cohort after 4 weeks of treatment.

In contrast, seven modules – M1.3 (B-cells), M1.5 (myeloid cells), M1.8, M3.2 (inflammation), M3.4, M3.6 and M3.9 – significantly decreased (*P* *<*8.5e-03) in expression in MTX good responders. Also, patients characterized as MTX non-responders demonstrated a significant decrease in the expression of module M1.1 (plasma cells). Significant changes in one of the 27 modules (M3.7) was observed in the MTX cohort between pre-treatment and 4 weeks, regardless of MTX treatment response at 6 months.

### Comparison of module change between the TNFi- and MTX-treated cohorts

When comparing module expression in the TNFi cohort, changes in expression were smaller in the MTX-treated patients; with fewer modules significantly changing in expression between pre- and post-treatment. Several modules (M1.3, M1.8, M2.3, M3.4, M3.6, M3.7 and M3.9) in the MTX cohort demonstrated a significant, but inverse change in module expression compared with the good responders to adalimumab (Figs 1 and 2) and the three independent US-recruited RA cohorts, previously described by Oswald *et al.* [13]. One of these modules was associated to B-cell biology (M1.3), and another to erythrocyte biology (M2.3). The same opposing direction of change in modular expression was observed in non-responders to MTX for module M1.1 (plasma cells) and M3.7.

### Module expression changed towards a disease-free state

To determine whether module expression transitioned towards a disease-free state, module expression was compared between TNFi-treated patients and disease-free controls. Disease-free controls were sex-matched 1:1 (Wilcoxon Signed-Rank Test, *P* = 0.79), but it was not possible to age-match controls and patients due to the nature of sample collection. For 22 of the 27 modules, difference in module expression between patients and controls was reduced after treatment, suggesting module expression transitioned in the direction of a disease-free state.

For six modules – M1.3 (B-cells), M1.5 and M2.6 (myeloid cells), M1.7 (MHC and ribosomal proteins), M3.3 (inflammation) and M3.4 – the difference in expression between pre-treatment TNFi samples and disease-free controls was significant (*P* <8.5e-03) at baseline, but not following treatment with adalimumab in both the good and non-responders, suggesting these modules were affected by adalimumab treatment, but not adalimumab treatment response.

Only one module – M3.2 (inflammation) – showed a statistically significant difference (*P* *<*8.5e-03) in expression in good responders *vs* non-responders to adalimumab. By 3 months, module M3.2 expression remained statistically and significantly different to disease-free controls (*P* *=* 0.041), while no difference was detected between good-responder patients and disease-free controls (*P* *=* 0.190), i.e. good responders to adalimumab had transitioned to a disease-free state whereas non-responders had not (Table 2).

**Table 2:** Transcript expression in the adalimumab cohort at baseline and follow-up was compared with disease-free controls

	Good-responders		Non-responders	
Module ID	Effect size	*P*-value	Effect size	*P*-value
BL 1.1	0.199	4.81E-07^*^	0.176	1.71E-05^*^
FU 1.1	0.119	4.42E-03^*^	0.078	0.019^*^
BL 1.2	−0.071	0.127	−0.118	0.124
FU 1.2	−0.003	0.960	−0.028	0.718
BL 1.3	0.299	1.01E-05^*^	0.309	1.79E-05^*^
FU 1.3	0.095	0.240	0.098	0.246
BL 1.4	0.113	1.23E-05^*^	0.064	0.006^*^
FU 1.4	0.089	4.69E-04^*^	0.049	0.029^*^
BL 1.5	−0.215	9.04E-06^*^	−0.236	1.60E-06^*^
FU 1.5	−0.077	0.111	−0.093	0.057
BL 1.6	0.137	8.59E-05^*^	0.114	0.002^*^
FU 1.6	0.094	0.017^*^	0.049	0.190
BL 1.7	0.066	4.61E-03^*^	0.087	1.28E-05^*^
FU 1.7	0.002	0.926	0.034	0.094
BL 1.8	0.145	4.09E-06^*^	0.114	3.13E-05^*^
FU 1.8	0.095	3.91E-03^*^	0.071	0.013^*^
BL 2.1	0.187	3.79E-05^*^	0.212	1.88E-05^*^
FU 2.1	0.121	0.019^*^	0.133	0.016^*^
BL 2.11	0.058	0.084	0.053	0.110
FU 2.11	0.121	0.019^*^	0.029	0.410
BL 2.2	−0.451	2.56E-06^*^	−0.391	1.94E-07^*^
FU 2.2	−0.261	1.03E-03^*^	−0.237	8.87E-04^*^
BL 2.3	−0.042	0.063	−0.155	0.145
FU 2.3	−0.056	0.023^*^	−0.177	0.089
BL 2.4	0.203	3.91E-08^*^	0.218	8.13E-09^*^
FU 2.4	0.105	1.88E-03^*^	0.121	6.28E-04^*^
BL 2.5	−0.063	2.52E-05^*^	−0.072	9.56E-06^*^
FU 2.5	−0.062	9.63E-05^*^	−0.072	5.98E-05^*^
BL 2.6	−0.233	1.14E-05^*^	−0.250	7.09E-06^*^
FU 2.6	−0.066	0.209	−0.068	0.207
BL 2.7	0.103	1.45E-04^*^	0.143	2.31E-05^*^
FU 2.7	0.055	0.0395^*^	0.079	0.011^*^
BL 2.8	0.231	1.03E-06^*^	0.255	9.02E-07^*^
FU 2.8	0.126	0.004^*^	0.140	3.66E-03^*^
BL 2.9	0.017	0.611	−0.048	0.880
FU 2.9	0.029	0.400	0.005	0.892
BL 3.1	−0.080	0.073	0.048	0.444
FU 3.1	−0.082	0.080	−0.062	0.931
BL 3.2	−0.183	5.06E-06^*^	−0.207	1.02E-07^*^
FU 3.2	−0.052	0.190	−0.079	0.041^*^
BL 3.3	−0.239	6.15E-06^*^	−0.236	4.24E-06^*^
FU 3.3	−0.080	0.112	−0.080	0.982
BL 3.4	0.100	0.004^*^	0.087	0.005^*^
FU 3.4	0.063	0.102	0.051	0.133
BL 3.5	−0.085	0.097	0.141	1.11E-05^*^
FU 3.5	0.056	0.245	0.075	0.007^*^
BL 3.6	0.092	0.002^*^	0.111	5.11E-04^*^
FU 3.6	0.070	0.025^*^	0.074	0.029^*^
BL 3.7	0.162	5.22E-10^*^	0.143	1.71E-08^*^
FU 3.7	0.106	8.12E-06^*^	0.090	1.05E-04^*^
BL 3.8	0.213	2.37E-06^*^	0.205	1.28E-06^*^
FU 3.8	0.148	0.003^*^	0.137	3.11E-03^*^
BL 3.9	0.129	2.67E-04^*^	0.119	5.46E-04^*^
FU 3.9	0.081	0.031^*^	0.076	0.039^*^

Module expression at baseline (BL) and 3 months (FU) was compared with healthy module expression by fitting linear mixed models. Significance indicated by

* (*P* *<*0.05).

The effect size shows the relative difference in transcript expression between patients and controls. A negative effect size reflects a higher level of module expression in the patients compared with the controls, and a positive effect size reflects higher module expression in the controls compared with patients.

## Discussion

The discovery of reliable biomarkers has been hampered by lack of replication in published studies. However, a recent study by Oswald *et al.* [13] reported consistent changes in modular gene expression in good responders to TNFi therapy across three independent US RA cohorts. In this current study, we aimed to determine whether modular changes in gene expression during early TNFi treatment were observed in UK patients and, by investigating a MTX-treated cohort, determine whether the effects were TNFi-specific.

We identified a subset of gene co-expression modules that demonstrated consistent and statistically significant changes in expression between pre- and post-treatment within TNFi-treated patients; however, most of these modules changed irrespective of treatment response. Six of these modules significantly transitioned towards a disease-free state due to the effect of adalimumab.

In MTX-treated patients, the expression of two modules (M3.2 and M2.1) significantly changed in the same direction as the TNFi cohort, suggesting non-drug specific changes. However, in patients characterized as a good responder to MTX, eight modules (M1.3, M1.8, M2.3, M3.4, M3.6, M3.7 and M3.9) significantly changed in the opposite direction to TNFi-treated patients and thus appear to be drug-specific effects. This was also seen in the non-responder cohort for module M3.7.

Overall, less consistent changes in module expression were observed in the MTX cohort compared with the TNFi cohort and the three independent RA cohorts reported by Oswald *et al.* [13] Only two modules (M1.5 related to myeloid linage and M3.2 related to inflammation) displayed significant and consistent changes in module expression in the MTX cohort, the TNFi cohort and the three independent cohorts studied by Oswald *et al.* [13]. Just one of the two modules (M3.2) was different in adalimumab responders *vs* non-responders.

The lack of consistency observed in the MTX cohort could be due to the earlier sampling time-point (4-week) compared with the 3-month follow-up in the TNFi-treated RA cohorts. Typically, RA patients will not have responded to treatment with MTX by 4 weeks, and will still be in an inflammatory state [19]. Therefore, we cannot exclude the possibility that a later sampling time-point of 3 months may have revealed more consistent changes in module expression for MTX. Furthermore, the modest changes in module expression observed in the MTX cohort may reflect less improvement in disease activity by 4 weeks, in comparison to the 3-month follow-up time point in the TNFi cohorts. However, disease activity measures were not available at the 4-week time point to confirm this. Alternatively, the smaller magnitude of change in module expression, and inverse trends in module expression observed in the MTX cohort, could be biologically revealing and some changes in module expression may be TNFi-driven. Additional research is therefore needed to determine the impact of sampling time point on potential drug-specific effects.

Findings from module M3.2 are potentially clinically interesting as responders to adalimumab statistically and significantly transitioned to a disease-free state whereas non-responders did not. Future studies could compare module expression with changes in CRP, an established measure of inflammation used to determine disease activity. Measuring the expression of module M3.2 in conjunction with CRP could provide an improved biological measure of response.

Here we investigated the extremes of response by focussing on EULAR good- and non-responders; however, EULAR grouping is based on the DAS28 that is made up of both objective (swollen joint count, blood marker of inflammation) and subjective (tender joint count and patient assessment of wellbeing) measures [20]. Our results support the argument for the need of a more objective measure of treatment efficacy as biological improvement towards a disease-free state was observed for some modules in both DAS28-derived good and non-responders. This could suggest clinical misclassification of response, though was minimized by using extremes of treatment response. Nonetheless, an objective biological measure of treatment efficacy would allow this to be explored. Alternatively, it reveals that the drug has an effect on gene expression independent of treatment efficacy. The modules that showed significant differences between good- and non-responders should be prioritized for mechanistic studies to understand how treatment effects are mediated.

The comparison between patients and disease-free individuals demonstrated a significant change in module expression for seven modules towards a disease-free state following 3 months of TNFi treatment. Of these seven modules, six modules transitioned towards the expression seen in disease-free controls in the group of TNFi non-responders, with only one module, module M3.2, transitioning more in good responders than non-responders.

Transcripts in module M3.2 are immune and inflammation related, relevant to RA pathophysiology, including genes involved in TGF-beta, TNF signalling, apoptosis and lipopolysaccharide biology [14]. Furthermore, genes in module M3.2, such as *NFKBIE*, *IRF2BP2*, *MAPKAP-K2, IL1B* and *IFRD1* map to the IFN type 1 signalling pathway [21–26]; a pathway linked to TNFi response in the RA literature [27–29]. Moreover, an enrichment of genes involved in IL-1, IL-17, IL-13, IL-4 and IL-10 signalling, plus genes involved in STAT3 modulation; a Th17 transcription factor, are in module M3.2 [30–32]. Further research is required to assess the potential of this module, or a combination of relevant modules, in predicting treatment response.

Other studies have identified gene expression modules in transcriptomics datasets derived from synovial tissue, which is also a heterogeneous population of cells. For example, Aterido *et al.* used a weighted correlation network analysis approach to identify co-expressed modules of genes in synovial tissue from RA patients treated with a TNFi [6]. In that paper, several modules, including a module enriched for genes related to nucleotide metabolism, were statistically associated with TNFi response; however, a direct comparison with the findings of the current study is not possible due to the different ways the modules were defined. In the Aterido paper, associations between gene expression modules and TNFi response were further corroborated using genetic datasets. In the future, it will be important to perform genetic analysis of gene expression modules defined by Chaussabel *et al.* However, this was beyond the scope of the current study.

A limitation of this and other similar studies is that gene expression data were derived from whole blood, therefore limiting the interpretation of pathway-specific changes in gene expression that rely on specific cell populations to drive changes in module expression. As the modules analysed here were originally defined in whole blood, and one of the aims of this study was to reproduce previously published findings by Oswald *et al.* [13], we chose to measure gene expression levels in whole blood samples. In the adalimumab-treated patients, we observed changes in modular gene expression in the same direction as those observed by Oswald *et al.* These findings therefore add to the evidence that whole blood transcriptomics analyses have the potential to identify important biomarkers of treatment response. Future analysis assessing changes in transcript expression in isolated cell populations would be useful to explore pathway-specific effects.

It was not possible to determine whether modular changes were solely driven by TNFi in the adalimumab cohort as the majority of adalimumab-treated patients were concomitantly treated with methotrexate (Table 1). Methotrexate was ineffective at controlling disease symptoms, thereby necessitating escalation to biologics, therefore it could be that the observed changes in modular expression resulted from a combined effect from methotrexate and adalimumab therapy.

A further limitation was that control data were generated separately to the patient data, resulting in a batch effect that was subsequently corrected for. However, as all data were treated identically, batch correction should not have affected the observed differences when comparing good- or non-responders to disease-free controls.

Lastly, a limitation of the analysis is that EULAR was measured at two different time points: 3 months in the adalimumab cohort and 6 months in the MTX cohort. We analysed the 3-month EULAR expression data for the MTX-treated patients, and in that analysis (Supplementary Fig. S1, available at *Rheumatology* online) we did observe module expression changed in the same direction as the 6-month time point, but these changes did not reach statistical significance. This was likely due to the 3-month response data being available for only 19 good responders and 22 non-responders to MTX. The fewer sample numbers meant this analysis had reduced statistical power.

A strength of this study is that the addition of control data enabled investigation of the direction of change in modular expression in the context of molecular health. Despite the low number of controls, significant and consistent changes in module expression, in the direction of a disease-free state, were observed.

Our findings provide additional evidence for the utility of transcriptomic data to develop prediction tests or monitor treatment pathways that are responsive to treatment in RA and potentially in other immune-mediated inflammatory diseases where TNFi are similarly effective.

## Conclusion

In summary, we have replicated changes in module gene expression that were observed by Oswald *et al.* in the adalimumab-treated patients [13]. Further research is needed to investigate the effect of sampling time point on modular response to MTX. Further well-powered studies of genes within module 3.2, a module that constitutes transcripts involved in inflammation, are also warranted for TNFi response classification and the potential use of module 3.2 for prediction modelling.

## Supplementary Material

keac204_Supplementary_Data

## Data Availability

Data are available upon reasonable request by any qualified researchers who engage in rigorous, independent scientific research, and will be provided following review and approval of a research proposal and Statistical Analysis Plan (SAP) and execution of a Data Sharing Agreement (DSA). All data relevant to the study are included in the article.
