# Supplementary material for: Pre-defined gene co-expression modules in rheumatoid arthritis transition towards molecular health following anti-TNF therapy
Source: Rheumatology (Oxford). 2022 Apr 4;61(12):4935–44. doi: 10.1093/rheumatology/keac204 (PMC9707314; doi:10.1093/rheumatology/keac204)

Supplementary Material

**Supplementary Table S1.** Number of probes per module that significantly changed in expression between baseline and follow-up (3-months) in the adalimumab cohort for good responders and non-responders to treatment. Significance was determined by conducting a student’s t-test (p < 0.05).

|  | |  | | Number of significantly changed probes | | | |
| --- | --- | --- | --- | --- | --- | --- | --- |
| Module ID (n) | **Probes per module (n)** | | **Change in expression** | **GR** | ***P* value** | **NR** | ***P* value** |
| 1.1 | 19 | | Increase | 13 | 5.14E-03 | 8 | 3.09E-03 |
| 1.2 | 154 | | Decrease | 56 | 2.22E-16 | 8 | 7.12E-03 |
| 1.3 | 100 | | Increase | 91 | 3.48E-41 | 56 | 1.28E-12 |
| 1.4* | 148 | | Increase | 56 | 1.66E-03 | 17 | 1.00E+00 |
| 1.5 | 228 | | Decrease | 145 | 1.32E-56 | 57 | 2.52E-19 |
| 1.6* | 47 | | Increase | 19 | 3.81E-02 | 3 | 2.42E-01 |
| 1.7 | 276 | | Increase | 131 | 4.36E-18 | 37 | 3.99E-12 |
| 1.8* | 202 | | Increase | 112 | 2.19E-14 | 15 | 2.93E-01 |
| 2.1* | 256 | | Increase | 110 | 3.04E-07 | 28 | 1.00E+00 |
| 2.11 | 186 | | Increase | 95 | 8.18E-04 | 33 | 4.90E-11 |
| 2.2* | 56 | | Decrease | 26 | 6.58E-10 | 5 | 5.69E-02 |
| 2.3 | 168 | | No change | 18 | 8.09E-01 | 7 | 1.00E+00 |
| 2.4 | 146 | | Increase | 110 | 8.16E-49 | 22 | 2.03E-07 |
| 2.5 | 285 | | No change | 67 | 7.95E-01 | 14 | 1.74E-01 |
| 2.6 | 230 | | Decrease | 177 | 1.55E-76 | 108 | 7.31E-38 |
| 2.7* | 37 | | Increase | 21 | 1.58E-03 | 5 | 3.58E-01 |
| 2.8* | 186 | | Increase | 126 | 5.83E-46 | 6 | 6.85E-01 |
| 2.9** | 200 | | Decrease | 120 | 5.85E-01 | 49 | 5.28E-12 |
| 3.1 | 152 | | No change | 32 | 8.52E-01 | 5 | 6.04E-02 |
| 3.2 | 372 | | Decrease | 212 | 6.52E-63 | 133 | 6.73E-39 |
| 3.3 | 336 | | Decrease | 233 | 1.89E-86 | 133 | 3.18E-43 |
| 3.4 | 382 | | Increase | 210 | 2.16E-17 | 50 | 1.40E-09 |
| 3.5 | 35 | | Decrease | 21 | 1.00E+00 | 3 | 2.39E-01 |
| 3.6 | 360 | | Increase | 217 | 4.62E-05 | 57 | 2.72E-07 |
| 3.7 | 371 | | Increase | 238 | 3.76E-55 | 59 | 1.11E-04 |
| 3.8* | 359 | | Increase | 220 | 1.36E-51 | 27 | 4.33E-01 |
| 3.9 | 297 | | Increase | 184 | 1.56E-21 | 40 | 7.04E-09 |

n= number of probes in module, NR= number of probes that changes in the non-responder group, GR= number of probes that changes in the good-responder group. *Significant change in good-responders but not in non-responders. **Significant change in non-responders but not good-responders.

**Supplementary Table S2.** Number of probes per module that significantly changed in expression between baseline and follow-up (4-weeks) in the MTX cohort for both good and non-responders to treatment. Significance was determined by conducting a student’s t-test (p < 0.05).

|  | | Number of significantly changed probes | | | |
| --- | --- | --- | --- | --- | --- |
| Module ID | **Change in expression** | **GR** | ***P* value** | **NR** | ***P* value** |
| 1.1** | Decrease | 3 | 2.27E-01 | 5 | 4.45E-02 |
| 1.2 | No change | 11 | 4.98E-01 | 5 | 1.00E+00 |
| 1.3* | Decrease | 25 | 7.14E-04 | 2 | 4.97E-01 |
| 1.4 | No change | 31 | 9.27E-02 | 5 | 1.00E+00 |
| 1.5* | Decrease | 6 | 9.04E-05 | 7 | 1.00E+00 |
| 1.6 | No change | 3 | 1.00E+00 | 2 | 1.00E+00 |
| 1.7 | No change | 31 | 1.00E+00 | - | - |
| 1.8* | Decrease | 22 | 2.18E-03 | 11 | 2.19E-01 |
| 2.1** | Decrease | 22 | 1.00E+00 | 4 | 2.05E-04 |
| 2.11* | No change | 19 | 1.38E-02 | 4 | 6.23E-01 |
| 2.2 | No change | 13 | 2.41E-01 | - | - |
| 2.3* | Increase | 21 | 5.04E-04 | 3 | 6.23E-01 |
| 2.4 | No change | 27 | 1.00E+00 | 13 | 1.00E+00 |
| 2.5 | No change | 2 | 3.73E-01 | 6 | 5.76E-01 |
| 2.6 | No change | 22 | 1.00E+00 | 4 | 2.15E-01 |
| 2.7 | No change | 12 | 4.91E-01 | 21 | 1.00E+00 |
| 2.8 | No change | 10 | 2.71E-01 | 10 | 1.00E+00 |
| 2.9 | No change | 45 | 5.67E-02 | 13 | 4.99E-01 |
| 3.1 | No change | 42 | 1.03E-01 | 1 | 6.22E-01 |
| 3.2* | Decrease | 39 | 3.87E-05 | 9 | 3.76E-01 |
| 3.3 | No change | 1 | 7.88E-02 | 6 | 7.52E-01 |
| 3.4* | Decrease | 35 | 4.59E-05 | 8 | 1.00E+00 |
| 3.5 | No change | 44 | 1.00E+00 | 13 | 1.00E+00 |
| 3.6* | Decrease | 27 | 3.48E-04 | 1 | 5.05E-01 |
| 3.7*** | Decrease | 43 | 2.13E-06 | 1 | 7.40E-03 |
| 3.8 | No change | 2 | 1.00E+00 | 2 | 1.00E+00 |
| 3.9* | Decrease | 3 | 2.26E-05 | 8 | 2.86E-01 |

NR= number of transcripts that changes in the non-responder group, GR= number of transcripts that changes in the good-responder group. Less than 1 transcript indicated by “-“.

*Significant change in good-responders only. **Significant change in non-responders only. ***Significant change in both responder groups.

**Supplementary Figure S1.** Fraction of probes per module that significantly changed in expression between baseline and follow-up (3-months) in the MTX cohort for good responders and non-responders to treatment. From left to right graphs show module 1.1 – 3.9. Red bars indicate good responders and green bars show non-responders to adalimumab. Direction of bars 0 to 1 indicate increase between pre-treatment and post-treatment and 0 to -1 indicate a decrease between pre-treatment and post-treatment. Label abbreviations: CTL; cytotoxic T lymphocyte. IFN; Interferon inducible. INFL; Inflammation.


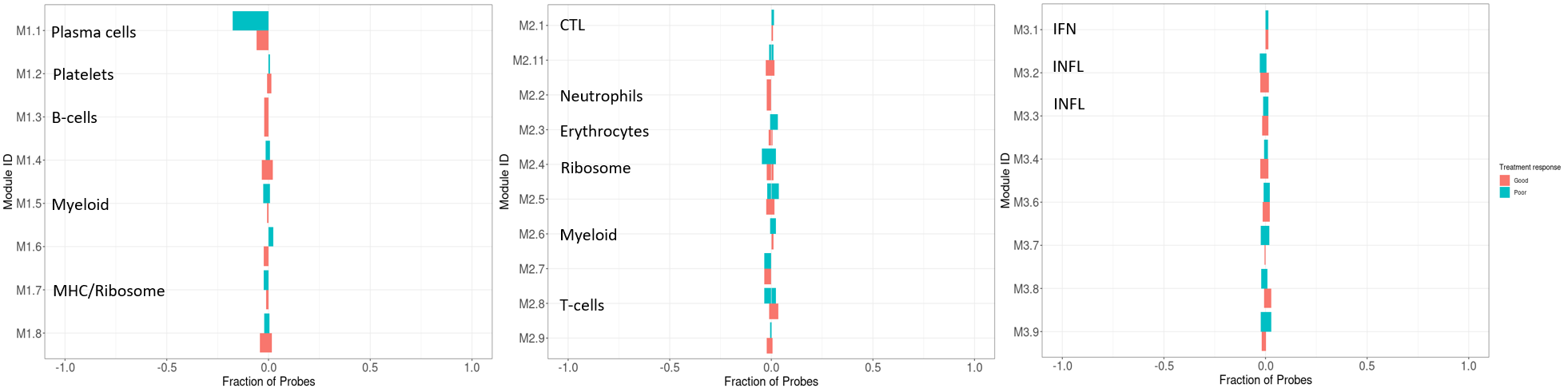

Supplement: keac204_Supplementary_Data [file keac204_supplementary_data.docx]
